# Cerebro-Spinal Arachnitis or Paralysis

**Published:** 1852-03

**Authors:** James Whitmire

**Affiliations:** Metamora, Ill.


THE NORTH-WESTERN

MEDICAL AND SURGICAL JOURNAL.

VOL. IV.] MARCH, 1852. [No. 6.

### Part I.—Original Communications.

ARTICLE I.

*Cerebro-Spinal Arachnitis or Paralysis.* By JAMES S. WHITMIRE, M. D., of Metamora, Ill.

In the N. W. Med. and Surg. Journal of Nov., 1851, there is an article written by Jas. Smick, M. D., of Menard Co., Ill., on one of those anomalous diseases of unmistakable Miasmatic origin, which he calls, or perhaps what the profession is pleased to call Cerebro-Spinal Arachnitis. But from what I have seen of this disease, I am disposed to consider it a partial *inertia* or *paralysis* of the Cerebro-Spinal system, from specific causes. The congested condition of the blood vessels and serum in the Arachnoid cavity, as sometimes found on post mortem examinations, may easily be accounted for on the principle of debility, from the want of h natural supply of nervous energy, instead of any direct irritating cause. Consequently the blood, forced into the enfeebled capilary vessels of the part, rendered so by the direct sedative influence of that insidious, protian poison (miasm) is retained, and the watery portions, with sometimes broken down globules, escape through the coats of the vessels as though a dead membrane, which accounts, in some measure at least, for the sanguino-cerous fluid sometimes found on post mortem examination in the Arachnoid cavity. In cases of sudden death from this disease, Dr. Smick supposes there is either congestion or total paralysis of the great nervous centers. Now from the above view of the pathology of this disease, death cannot occur with both conditions being present in a greater or less degree. And in cases that are more mild in the commencement, with a constant repetition of the same cause without appropriate treatment, would terminate in death in a few days from the same cause, and that too without any other signs in post-mortem examination than an atonic congestion and perhaps a small quantity of serum that has made its way, as it were, through a dead membrane. I am now speaking, and so wish to be understood throughout all of those cases that are clearly referable to a miasmatic cause. For no one could imagine a case of Meningitis proper, without an active state of inflammation and tonic congestion; and and there is no one so presumptuous, except Hydropathy or the little pill faculty, who would dare treat it without active depletion and other antiphlogistic remedies. It will therefore be understood there is, in my opinion, no inflammation whatever, and the signs that presented themselves on post-mortem examination were not effusion from inflammation, but an oozing of bloody serum through the unnaturally distended and paralyzed meningeal capillary vessels. That this anomalous Cerebro-Spinal Miasmatic disease exhibits its own peculiar class is undoubted, and Dr. Smick has given them very faithfully; and though I have never seen an erysipelatous inflammation follow an abatement of the symptoms, yet I can easily imagine that the condition of the system would be favorable to its developement. And though my experience in the treatment of this disease, has in no wise been as extensive as Dr. Smick's, yet it has been far more satisfactory, acknowledging at the same time that I never witnessed the disease in an epidemic form, my cases all being sporadic and at from three to nine months intervals. Since 1845, there has ten cases occurred in my practice, eight of which were distinctly marked cases, and exhibited the worst form of the disease; seven of them recovered, and one died.

*Case* 1*st.* Miss M. E., aged 16 years, was taken with violent pain in the head, preceded with a chilly sensation. She remained for three or four hours delirious, after which she become quite rational, but much indisposed during the night. The father being away from home, I was not called till next day, Oct. 8th. She was taken 11 o'clock, one hour earlier than the day previous. I saw her 2 o'clock P. M. She was tossing herself back and forth on the bed; layed mostly on her back, her head and feet remaining pretty much in the same place, her head being shoved down into the bed so as to almost make a complete half circle from head to feet. Pulse 100 and small, countenance pale, surface cold, extremities particularly. Talked incoherently, or rather a moaning, grumbling noise, with paralysis of the muscles of deglutition, so that it was impossible to administer medicine by the mouth. Ordered a Turpentine enema in starch with salt to evacuate the rectum; after which Sulph. Ether in starch was administered per anum: hot turpentine cloths applied to spine, breast and bowels, dry friction with soft flannel and ground mustard to the extremeties, alternating with bottles of hot water. In two hours of diligent employment of these remedies, reaction began gradually to come on; in another hour she talked and swallowed, but complained of violent pain in the head, and her countenance began to be quite flushed. I opened a vein and took about 10 ℥ of blood, which gave immediate relief. I then ordered a blister to the spine and back of the neck, gave an active dose of Calomel and Jallap, and put her upon minute doses of Tart. Antimon., during the night applied cold to the head, &c., &c., mistaking it for a case of Cerebro-Spinal Meningitis. The blister done well, bowels moved two or three times freely, and she remained quite comfortable 9 o'clock the next day, which was about an hour sooner than her paroxysm the day previous, when all the former symptoms returned with redoubled violence. I was immediately summoned to see her, and when I arrived she was in a state ot Articulo Mortis, there never having been the slightest attempt of nature at reaction. This case being the first that I had ever witnessed, and I a young practitioner, it never once occurred to me that it might be from any other cause than inflammation ot the meninges of the brain, till to my great surprise and chagrin I walked into the house and found her dead. Terminating as it did, and being strictly periodical, led me to decide on a different course of treatment the next case that came under my care, let the consequences be what they might; for it was morally certain that it could be no worse than the other, and it had done me no particular credit in the immediate neighborhood.

*Case 2d.* Mr. F., aged 30, of full habit and sanguine temperament, was taken March 20th, 1848, at 12 o'clock M. I saw him 4 o'clock P. M. He was in a complete state of insensibility, tossing himself from one side to the other; respiration hurried to about 40 in a minute; the whole surface cold; countenance pale; back in a tonic curve; pulse 100, and very small; eyes set back, mouth firmly closed, and could not be forced to attempt to swallow even water. This patient had been unwell for two or three weeks, and though able to go about his ordinary business, yet his usual hilarity of spirits were gone, and he expressed himself as feeling miserable, but not sick, and for two or three days previous to the attack had complained of a general feeling of fullness and pain in the head with occasional double vision. As nothing could be given him by the mouth, I ordered a stimulating enema of

Turpentine, Molasses and Salt to evacuate the rectum; after which Laudanum and Sulph. Ether in starch was given per anus; applied hot turpentine cloths to the spine, breast and bowels; dry friction with soft flannel and dry mustard to the extremities, bottles of hot water, &c. In an hour he become more quiet, and the surface began to assume a more natural warmth. In an hour more he was quite comfortable, and began to question us in relation to the cause of our presence, and why so many of us were there, &c. He still complained of violent pain in the head, and his face began to be flushed, and his pulse had risen to 110 and increased in volume, but not in force. I then took 10 ℥ of blood from the arm, which seemed to relieve the pain in his head for the present, but he soon become restless and wandering and disconnected in his thoughts. Ordered blister to the spine from the inferior dorsal vertebra to the ociput, and gave five powders, each consisting of 3 grs. Calomel, 10 grs. Camphor, 10 grs. Quinine, ⅛ gr. Morphia, one to be taken every two hours till next day, (21st,) when I saw him, at 3 o'clock P. M. Found him quite comfortable; head clear except a little buzzing from the effect of the quinine. Calomel had moved his bowels, blister drew well, pulse 90 and soft. Ordered six powders, each containing 3 grs. Quinine, 5 grs. Camph., ½ gr. Morph., one to be taken every four hours till next day, when I saw him for the last time professionally. Found him decidedly convalescent. Ordered wine and peruvian bark three times per day for three weeks, and what moderate exercise his strength would permit him to take. Recovered perfectly. I am now perfectly satisfied that venesection done this man an injury for the time, though if I had waited one or two hours longer he might have borne it better.

*Case 3d.* M. B., a young lady aged 17 years, of a sanguine billious temperament, florid complexion, &c. Was taken Dec. 10th, 1851, with an ague, as the family supposed

Complained of chilliness; pain in the head, with an uneasy sensation or stricture across the chest, accompanied with difficulty of breathing; frequent sighing and occasional involuntary coughing; but the symptoms gradually wore away, so that in the course of two or three hours she was able to be up and attend to her domestic concerns. The next day she was taken again about the same hour, but much more violently. I was immediately sent for, but having about ten miles to ride I did not see her till 6 o'clock P. M.---at least six hours after the commencement of the paroxysm; had been speechless and unconscious ever since. Found her writhing in apparent agony; pulse 120, small; surface cold, countenance pale, and would swallow anything you would put to her lips. Her head was drawn back almost between the shoulders; occasional severe spasms of the extremities, alternating with spasmodic action of the muscles of respiration, so that she would make forty attempts in a minute to take a full inspiration, but would always be cut short by the ungovernable spasmodic action of the muscles. Gave her Tinct. Opii and Sulph. Ether freely; ordered hot turpentine friction, &c., as in former cases. In twenty minutes after the administration of the Tinct. and Ether the irregular action of the respiratory muscles began to subside, and in three hours more she was quiet, though yet quite unconscious, save when you would speak in a very sharp tone to her, and then she would seem to notice for a moment only. I now determined to try the anti-periodic treatment without any depletion save by the bowels. Ordered a blister to the spine and back of the neck; six powders, each consisting of 3 grs. Calomel, 8 grs. Quinine, 8 grs. Camphor, $\frac{1}{8}$ gr. Morphine, one to be given every two hours till gone. Next day (12th) I saw the patient at 4 P. M., three hours after the time for the occurrence of the paroxysm, and she was quite rational. Head clear of pain, but complained of much soreness and muscular debility, which was, no doubt, occasioned by their spasmodic action the day previous. Bowels had moved freely ; gave her seven more powders, each containing ⅛ gr. Morphine, 3 grs. Camphor, 3 grs. Quinine. Her pulse was 85, full and soft. She was to take her powders every four hours till gone; after which she took wine and bark three or four times per day for a week. The convalescence of this patient was more speedy than case 2d, and her subsequent health from all present appearances, bids fair to be as good as she previously enjoyed.

The other seven cases I treated previous to the last, all on the same principle as case 3d, and with at least as satisfactory results, only thinking it proper to bleed in one case. And now, if this peculiar disease is accompanied with either primary, or secondary inflammation, I am at a loss to know why it is, that my patients are yet living. For certainly the *Vis Medicatri Natura* must have been powerful to overcome both medicine and disease. For it is a statistical fact, that at least one out of three under the Antiphlogistic treatment dies. That circumstances might occur, in which I would bleed, I can easily imagine ; yet I have never had occasion to use the lancet but once since case 2d ; and the principal objection I have to Dr. Smick's treatment is, that he runs too great a risk of loosing his patient in the second paroxysm, on account of not giving Quinine sooner. And I am more and more convinced of the justness of Dr. Smick's remark, that "there is no remedy that requires more caution in using it, than bleeding in Cerebro-Spinal Arachnitis." And from my own observation, I think the disease is more likely to assume a Typhoid character, from at least indiscriminate antiphlogistic measures, though that tendency has not presented itself in my practice. My opinion, however, can only go, in an intelligent profession, for what it is worth, for these are days of progress, and every thing that is presented to the public is scanned and sifted before it is acknowledged ; and if there was less fear among ourselves of controversy, we might do better for our patients too.

P. S. I am not personally acquainted with Dr. Smick, but I trust I have said nothing to offend, as it has not been in that kind of spirit I have written, but rather to invite some more of the profession to speak out.

